# A Review of Safety and Design Requirements of the Artificial Pancreas

**DOI:** 10.1007/s10439-016-1679-2

**Published:** 2016-06-28

**Authors:** Helga Blauw, Patrick Keith-Hynes, Robin Koops, J. Hans DeVries

**Affiliations:** 1 Department of Endocrinology, Academic Medical Center, University of Amsterdam, P.O Box 22660, 1100 DD Amsterdam, The Netherlands; 2 Inreda Diabetic BV, Goor, The Netherlands; 3 TypeZero Technologies, LLC, Charlottesville, VA USA; 4 Center for Diabetes Technology, University of Virginia, Charlottesville, VA USA

**Keywords:** Diabetes, Closed loop system, Medical device development, Risk analysis, Sensors

## Abstract

As clinical studies with artificial pancreas systems for automated blood glucose control in patients with type 1 diabetes move to unsupervised real-life settings, product development will be a focus of companies over the coming years. Directions or requirements regarding safety in the design of an artificial pancreas are, however, lacking. This review aims to provide an overview and discussion of safety and design requirements of the artificial pancreas. We performed a structured literature search based on three search components—type 1 diabetes, artificial pancreas, and safety or design—and extended the discussion with our own experiences in developing artificial pancreas systems. The main hazards of the artificial pancreas are over- and under-dosing of insulin and, in case of a bi-hormonal system, of glucagon or other hormones. For each component of an artificial pancreas and for the complete system we identified safety issues related to these hazards and proposed control measures. Prerequisites that enable the control algorithms to provide safe closed-loop control are accurate and reliable input of glucose values, assured hormone delivery and an efficient user interface. In addition, the system configuration has important implications for safety, as close cooperation and data exchange between the different components is essential.

## Introduction

For many patients with type 1 diabetes it is difficult to maintain normal blood glucose levels with the currently available therapies, which include multiple daily insulin injections or continuous subcutaneous insulin infusion with or without the use of a continuous glucose monitor.^6^ These therapies are all patient-managed; the patient has to make treatment decisions multiple times per day to control his blood glucose and it requires a substantial commitment from the patient in order to reach treatment goals. Multiple factors affect blood glucose and both excessively high and low glucose levels have negative health effects. An artificial pancreas can assist the patient in overcoming these problems by taking over glucose control from the patient in certain situations or even 24 h per day.

**Figure 1:**
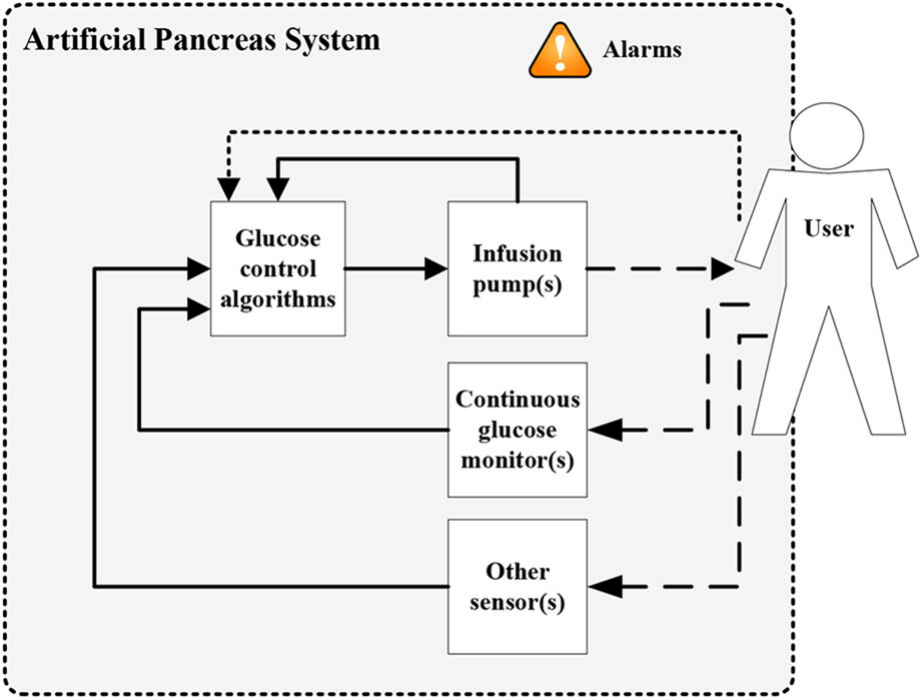
Diagram of the artificial pancreas system containing the three main components, optionally other sensor(s), and alarms. The user is the patient who can interact with the whole system and, if included in the system, announce meals to the control algorithms, as represented by the dotted lines. Solid lines indicate signals and communication between the components. Dashed lines starting from the user represent physiologic measurements and the dashed line to the user indicates the pump action.

The main components of an artificial pancreas are a continuous glucose monitor to assess blood glucose concentration, a set of glucose control algorithms to calculate the amount of insulin needed, and an infusion pump for insulin administration to lower blood glucose. Figure 1 shows a diagram of the artificial pancreas system as discussed in this review article. There is not just one artificial pancreas, as the implementation of these components can differ and more components can be added to the system. Over the past decade multiple research groups and companies have worked to develop artificial pancreas systems, with promising results in clinical studies.^23^ At this moment, however, there is still no artificial pancreas system available on the market. The prototype systems that have been used in clinical studies are not yet suitable for daily and independent use by patients.

As clinical studies with artificial pancreas systems move to unsupervised real-life settings, product development will be a focus of companies over the coming years. A crucial aspect in the development and approval of medical devices is patient safety. Because the artificial pancreas aims to automate blood glucose control, which can lead to severe health damage in case of malfunction, its design must meet stringent safety requirements. Beside clinical safety requirements stated by the Food and Drug Administration (FDA),^72^ there are currently no directions or requirements regarding safety in the design of an artificial pancreas. To promote patient safety and facilitate the development of artificial pancreas systems for daily use, this review aims to provide an overview and discussion of safety and design requirements of the artificial pancreas.

## Methods

A structured literature search was performed using PubMed after identification of the MeSH terms and free-text terms, for search in title and abstract, relating to the three search components: type 1 diabetes, artificial pancreas, safety or design. The search query was: (“diabetes mellitus, type 1”[MeSH Terms] OR “type 1 diabetes”[tiab]) AND (“pancreas, artificial”[MeSH Terms] OR “artificial pancreas”[tiab] OR “bionic pancreas”[tiab] OR “closed-loop”[tiab]) AND (“safety management”[MeSH Terms] OR “patient safety”[MeSH Terms] OR “equipment safety”[MeSH Terms] OR “equipment failure”[MeSH Terms] OR “risk management”[MeSH Terms] OR “device approval”[MeSH Terms] OR “equipment design”[MeSH Terms] OR safe[tiab] OR safety[tiab] OR “risk management”[tiab] OR approval[tiab] OR “device design”[tiab] OR failure[tiab]). The search was performed on December 15, 2014, was updated on April 22, 2015 and March 24, 2016 and resulted in 187 articles, of which 19 articles were excluded based on the title and 16 on the abstract. The excluded articles were not about the artificial pancreas for treatment of type 1 diabetes, were not written in English or were news articles. The remaining articles were read to identify safety issues and design requirements for the artificial pancreas. Subsequently, reviews or other articles that did not add relevant findings or recommendations compared to more recent reviews, and clinical studies or in silico studies that assessed safety but without relevant findings were excluded (95 articles). In addition, 22 articles were selected from reference lists of included articles or the authors’ personal databases. The discussion of the safety issues and design requirements was extended with our own experiences in developing artificial pancreas systems.

## Background

### The Artificial Pancreas

The goal of an artificial pancreas is to achieve adequate mean blood glucose levels and stabilize blood glucose by limiting excursions, while limiting the occurrence of hypoglycemia and hyperglycemia. The adequacy of a patient’s mean blood glucose levels is assessed with the glycated hemoglobin (HbA1c) level. The American Diabetes Association recommends a HbA1c level below 7% (53 mmol/mol) for adults with type 1 diabetes, which corresponds to a mean glucose value below 8.6 mmol/l.^1^ To reach a mean glucose level below 8.6 mmol/l, intensive insulin therapy is required. However, for most patients it is difficult to anticipate changes in glucose, which is affected, among other things, by insulin dosage, meals and exercise under varying physical and environmental circumstances and it is not practical for patients to constantly monitor their glucose level and react on it. Furthermore, to avoid hypoglycemia, patients typically prefer slightly hyperglycemic glucose levels over low-normal values,^27^ especially before the night or long-term activities. For an artificial pancreas it is possible to continuously monitor the glucose level and adjust insulin dosing, which enables glucose control toward low-normal values 24 h per day.

The main components—continuous glucose monitor, set of control algorithms, insulin pump—are part of each artificial pancreas system, but the degree of automated glucose control is different. The different systems described here are adopted from the FDA guidance for the development of artificial pancreas systems.^72^ The first step toward fully closed-loop glucose control is a Threshold or Low-Glucose Suspend (LGS) system, which is patient-managed therapy. With this system the basal insulin infusion is reduced or suspended when the algorithmic blood glucose estimate reaches or approaches a low glucose value in order to prevent or reduce the severity of hypoglycemia.^9^,^48^ The next step is a Control-to-Range (CTR) system. This system not only reduces the rate of insulin infusion in case of low glucose values, but also may increase insulin dosing if a high glucose value is reached or approached. Between the predetermined low and high glucose value the insulin infusion is not affected by the sensor glucose values.^29^ Since the patient has to monitor his blood glucose, set basal insulin rates, and give pre-meal insulin boluses, glucose control with a CTR system is still supervised by the patient. The next logical step is a fully-automated Control-to-Target (CTT) system, which provides closed-loop glucose control by steering the glucose value towards a target level. With a CTT system patients do not have to monitor their blood glucose, but they may have to calibrate the continuous glucose monitor. In addition, hybrid CTT systems exist which are not fully closed-loop systems, because these systems require some input from the patient about meals. Both CTR and CTT systems can be insulin-only or bi-hormonal. A bi-hormonal artificial pancreas uses a second infusion pump to administer a second hormone such as amylin or glucagon as an additional means to control blood glucose levels. A gradual step toward continuous closed-loop glucose control is evening and overnight closed-loop control at home, which may already substantially impact glucose control as the night period can be hard to manage for patients.^42^,^44^,^52^

In this review, the term artificial pancreas refers to glucose control systems that require minimal intervention by the patient. Therefore, LGS systems fall outside the scope of this review.

### Regulatory Approval

Before patients can use an artificial pancreas in daily practice, the system has to be approved as a medical device by the applicable regulatory authority. For brevity, we only consider the regulatory approval in the United States (U.S.) and the European Union (E.U.). In the U.S. FDA approval is required and in the E.U. a Notified Body has to provide the CE-mark. Numerous insulin pumps and continuous glucose monitors, and one LGS system are approved and available on the market in the U.S. and E.U. At this moment no CTR or CTT systems are approved worldwide.

For the European regulation of medical devices the artificial pancreas is classified in class IIb. Class IIa, IIb or III require that a Notified Body assesses that the medical device and its quality system are in conformity with the requirements of the Medical Device Directive, which concern safety of the patient and other persons and the intended performance of the device. The company is free to choose any of the around 70 Notified Bodies. All Notified Bodies are assessed by their national Competent Authority, usually (an agency within) the ministry of Health, to ensure that they remain qualified for issuing the CE-mark. Once a CE-mark is obtained for a medical device, the company can market the device in all countries of the E.U.

The FDA regulates artificial pancreas systems as class III device systems.^72^ Class III is the highest medical device category and includes devices with high potential risk of injury and devices that are not found to be substantially equivalent to already marketed devices. Class III requires that the artificial pancreas developer submits a Premarket Approval (PMA) application. To get FDA approval, the PMA has to demonstrate that the artificial pancreas is safe and effective for its intended use. Generally, this will require data from clinical studies. Before a clinical study can be started, an Investigational Device Exemption (IDE) needs to be approved by the FDA.

In 2012, the FDA issued a guidance document with recommendations on the content of IDE and PMA applications for artificial pancreas systems.^72^ This guidance document followed after the FDA appointed the artificial pancreas as a Critical Path Opportunity in 2006. The Critical Path Initiative of the FDA aims to transform medical product development and evaluation in the U.S. in order to facilitate pre-market approval and promote innovation. The need for such transformation is illustrated by the approval of a LGS system: the FDA approved this system in 2013, whereas CE-mark was already obtained in 2009.^65^ Although the FDA guidance contains valuable information on how to demonstrate safety—especially on documentation and clinical evaluation—it does not cover design issues important for the safety of artificial pancreas systems.

### Risk Management

Risk management is part of both the CE-marking and FDA approval process to ensure and demonstrate that the design of an artificial pancreas is safe. Risk management is also required for the production process and clinical studies. The international standard for risk management of medical devices is ISO 14971. This standard provides a framework to identify hazards associated with the medical device, estimate and evaluate the risks associated with these hazards, control these risks, and monitor the effectiveness of that control. Together with the user requirements, the risk management should form the basis of the design of an artificial pancreas and it has to be kept up to date during the whole product lifecycle to ensure safety for the patient.

Since the artificial pancreas is a combination of different components, potential hazards will depend on these components. For the artificial pancreas system as a whole, the main hazards are over- and under-dosing of insulin and also of glucagon or other hormones in case of a bi-hormonal system. These hazards may cause severe hypoglycemia, severe hyperglycemia or diabetic ketoacidosis. The clinical requirements for safety are that the incidence of these events should not be increased by the artificial pancreas.^18^,^72^ Because of the combination of different components and glucose control algorithms within an artificial pancreas it may be difficult to assess safety of the composite system without significant clinical testing. Some of the particular risks known to be related to insulin pumps and continuous glucose monitors may be decreased, but also new safety issues related to network effects may emerge.^57^,^65^ *In silico* simulation of the operation of the entire device network may be useful in uncovering potential safety issues and speeding regulatory approval.^16^,^21^ Furthermore, an artificial pancreas is intended to be used by patients in daily life and not by health care professionals in a predictable environment. Therefore, user-related risks should be carefully evaluated and controlled. The safety issues and control measures described in this article, and summarized in Tables 1 and 2, should be considered in the risk management of an artificial pancreas.

## Safety of the Artificial Pancreas Components

### Continuous Glucose Monitor

Accurate glucose measurements are essential for the safety and efficacy of the artificial pancreas. Since regular glucose input is needed for the control algorithms, a continuous glucose monitor has to be part of the artificial pancreas system. Although a variety of new technologies such as implantable glucose sensors are under development, at this moment only subcutaneous enzyme glucose sensors with a coupled transmitter for wireless data transmission are practical for this purpose. These sensors generate a current proportional to the local glucose concentration and through a calibration procedure this current is converted into an estimated blood glucose value. Although currently not approved as a blood glucose reference for insulin dosing, the accuracy and reliability of enzyme glucose sensors continues to improve.^58^,^69^ Large positive sensor deviations from the true glucose value increase the risk of hypoglycemia, whereas sensor under readings increase the risk of hyperglycemia, because of inappropriate insulin delivery.^8^,^70^ In addition, glucagon was less effective in the prevention of hypoglycemia when delivery was delayed because of positive sensor deviations in a bi-hormonal artificial pancreas study.^13^

It is important to note that continuous glucose monitors produce a *time series* rather than a sequence of independent measurements. Although glucose sensor accuracy is often evaluated using the mean absolute relative difference (MARD) between sensor glucose values and paired reference glucose values, researchers have pointed out that MARD analysis underestimates the amount of useful information available within glucose sensor data. A recent assessment indicates that an MARD of <10% should be sufficient to use continuous glucose monitor values as a reference for manual insulin dosing.^40^ Recent head-to-head comparison studies of currently available continuous glucose monitors found overall MARDs (SD) of 12.2 (12.0)% and 19.9 (20.5)% at home,^43^ and of 12.3 (12.1)%, 10.8 (9.9)%, and 17.9 (15.8)% in an artificial pancreas study.^20^ Although absolute relative differences around 12% are acceptable for closed-loop glucose control, the occurrence of large errors is problematic for safe glucose control.^46^,^76^,^77^ Furthermore, the accuracy of enzyme glucose sensors is less during hypoglycemia compared to eu- and hyperglycemia.^43^,^46^

Several factors contribute to sensor inaccuracy, of which the most known factors are discussed here. The first factor is calibration error. Calibration of the glucose sensor is negatively affected by incorrect estimation of the background current of a sensor, by the use of an inaccurate reference glucose measurement or if calibration takes place during low, high or rapidly changing glucose values.^15^ A second factor is sensor delay, which is partly physiologic and partly inherent to the sensor itself and data processing.^15^ Thirdly, both slow and transient sensor drift can lead to sensor inaccuracy.^8^ Biofouling may reduce sensor output over time, whereas acute inflammation affects the accuracy during the hours after insertion of the sensor.^15^ Pressure-induced sensor attenuation may reduce sensor readings for 15–30 min, which mainly occurs overnight.^8^

As stated, the accuracy of currently available continuous glucose monitors differs and which sources of sensor error can be mitigated to increase accuracy and reduce the incidence of large errors will also be different. In general, we recommend that the overall MARD should be 15% or less with the sensors calibrated with self-monitored blood glucose (SMBG) values.^34^ Glucose sensor performance has to be assessed both in the clinical research center and at home using standardized procedures and multiple analysis methods.^43^ Evaluation of these results should result in identification of situations in which the accuracy is reduced and list the incidence of moderate (absolute relative difference ≥20%) and large (absolute relative difference ≥40%) sensor inaccuracies. Implications of these findings for safety of the glucose control have to be described together with appropriate measures to mitigate these risks.

Our personal recommendations for measures to reduce inaccuracies due to calibration error are: (1) base the decision to calibrate on the difference between a reference SMBG and the glucose sensor value and not on a predefined time period and (2) only allow calibration in case of euglycemia and stable glucose values or at least warn the user of the risk of a calibration error. The first measure is recommended because SMBGs have their own inaccuracy, both device and user related, that negatively influences sensor accuracy.^34^,^65^,^72^ Calibration of an acceptable accurate sensor with a SMBG may lead to a decrease of sensor accuracy. Therefore, if a SMBG and sensor value are within each other’s accepted error margins, recalibration should not be performed. If the SMBG and sensor value deviate from each other, it is necessary to take a second or even a third SMBG to reduce the probability that an erroneous SMBG is being used for calibration. The artificial pancreas software should be able to determine if one of the three performed SMBGs is an outlier and thus should not be used for calibration. In addition, the artificial pancreas should inform the patient when such a reference SMBG to assess agreement of the glucose sensor has to be performed, for example if a predetermined period (up to 24 h) after the last SMBG has passed and the glucose values are in the normal range and stable, or if two glucose sensors deviate from each other. Arguments to include two (or more) glucose sensors in the artificial pancreas system are discussed in the next paragraph. The second measure reduces the inaccuracy due to uncertain estimates of background current and sensor delay. These estimates should be based on careful evaluation of study results and be included in the calibration algorithm.

Both artificial pancreas systems with one glucose sensor 30,35,47,52,61,64 and systems with two glucose sensors 36,50,54,74 have been used in clinical studies. At this stage, there is no agreement on whether or not a second sensor is necessary for safety, or whether it is impractical to include a second sensor in the system. One reason to include a second sensor is that unnoticed inaccurate sensor readings may affect glucose control during a substantial period, as SMBGs may only be performed every 12 h (or even up to 48 h); this particularly affects the risk of hypoglycemia.^34^,^46^,^65^ Averaging multiple sensors can improve accuracy and especially reduce large sensor errors, but may also pose the risk of including inaccurate sensor readings in the blood glucose estimate.^14^,^76^ Also other (additional) strategies are possible to improve accuracy, such as selection of the most accurate sensor 15 and continuous detection of sensor deviations.^32^,^54^ Secondly, if a sensor that is run out or has failed is replaced it takes hours before the measurements of the new sensor are stable and reasonably accurate,^15^ in our experience even over 12 h. During these warm-up hours automated glucose control would not be possible if only one sensor is used, which affects safety. Thirdly, a second sensor provides a back-up in case of loss of communication with the other sensor. Communication aspects of the artificial pancreas system are discussed in “Combining the Components” section.

Apart from the mentioned measures, sensor accuracy can be improved with different software measures and a combination of measures will be required to address known factors that contribute to inaccuracy.^28^ In any case, an artificial pancreas should contain measures that enable detection of sensor inaccuracies and failure.^8^,^18^ Alarms should be given to the patient to check the glucose sensor in case of inaccuracies or the connection in case of lost communication, and to promptly replace the sensor if it fails. Persistent (more than 10–20 min) loss of sensor glucose values should result in safe transition to a fallback therapy, e.g. return to patient specific insulin basal rates.^8^,^53^

### Other Sensors

Beside continuous glucose monitoring, other sensors (e.g. heart rate or skin impedance sensors) can be included in the artificial pancreas system with the aim to measure physiological parameters that (indirectly) affect or reflect glucose control.^73^ In a review about physiological input for artificial pancreas system, Kudva *et al.* suggest to systematically determine efficacy and safety of including the various possible physiological parameters into glucose control algorithms.^45^ For the physiological parameters that can be directly or indirectly measured with a sensor, not only safety of the adaptation of the control algorithms is important, but also safety issues regarding the measurement method itself have to be considered. Just as for the glucose sensor, accuracy is the main issue because this may lead to over or under correction of hormone delivery. Accuracy requirements will depend on how much influence the measured parameter can have on the control algorithms. The availability of the measurement, which may be affected by communication between devices or compliance of the patient, also needs to be assessed. It needs to be demonstrated that in cases where the measurement is not available, but the targeted physiological phenomenon does occur, closed-loop glucose control is still safe.

Besides meals, exercise is considered to be the main challenging perturbation of glucose control in daily life.^8^,^70^ The influence of exercise on blood glucose depends on multiple factors related to the patient, the exercise and the environment and is therefore difficult to include in closed-loop glucose control.^19^,^45^ Unannounced exercise was related to hypoglycemia in a clinical trial in twelve adolescents using closed-loop basal insulin delivery 24 and an in silico trial with 100 virtual patients receiving basal insulin infusion.^67^ The authors from both studies indicate that exercise announcement well before exercise will be needed to reduce the insulin delivery in time to prevent exercise-related hypoglycemia, because of the delayed action of subcutaneously infused insulin. Safety concerns of manual announcements to the artificial pancreas include compliance and the difficulty of knowing whether the patient actually did what he announced. Including a sensor to measure exercise will not enable reducing insulin infusion before exercise, but it can be used to reduce insulin infusion during and also after exercise, as exercise also influences glucose concentrations several hours after exercise.^22^,^45^ Especially for exercise performed before the evening this may be an additional measure to reduce the risk of hypoglycemia.^51^ Sensors that are being investigated to measure exercise for closed-loop glucose control include accelerometers, heart rate and temperature sensors. In general, a sensor to measure exercise should only be included into an artificial pancreas if it reduces the risk of hypoglycemia.^19^

### Glucose Control Algorithms

The brain of the artificial pancreas consists of the algorithms that control the patient’s blood glucose concentration. This set of algorithms has to take over the glucose management from the patient and is the truly innovative component of the artificial pancreas. Therefore, research groups around the world have been focusing on the development of effective and safe glucose control algorithms. Many different control algorithms have been designed and evaluated, most of them being model predictive control, proportional-integral-derivative control, or fuzzy logic control.^23^ Evaluation of control algorithms is now successfully moving from supervised clinical research centers, and supervised out-of-hospital settings to unsupervised overnight use.^70^ Effective and safe automated glucose control during uncontrolled real-life situations, including irregular food intake, alcohol, stress, exercise and all kinds of spontaneous activities, will be the next step and challenge for the control algorithms.

The delayed action of subcutaneously infused insulin is the main difficulty for glucose control algorithms. Pharmacodynamic action of rapid-acting insulin peaks roughly around 90 min and action may persist up to 8 h. Insulin pharmacokinetics was found to have substantial variability between patients.^7^ Furthermore, the insulin sensitivity of a patient may vary due to several factors, which act on different time scales (from hours to years).^33^,^45^,^68^,^75^ Another aspect that has to be considered when designing control algorithms is the inaccuracy of the continuous glucose monitor, especially at lower and rapid changing blood glucose values.

Irrespective of the type of control, algorithms have to be developed using design requirements tailored to the target population, its environment and treatment goals.^23^ At this moment, it is not possible to design algorithms that include all relevant situations and parameters that influence or are influenced by glucose concentration, insulin, and glucagon sensitivity.^45^ Therefore, glucose control algorithms have to be responsive to changes in glucose trends and compensate for short term (timescale of hours) changes in insulin sensitivity at all times. The time interval at which the control algorithms determine the output should normally be on the order of 15 min, with each incoming glucose measurement being used to update the system estimate. Individualization of the control algorithms is needed to account for insulin sensitivity, but probably also for glucagon.^45^ Strategies to estimate insulin sensitivity include amongst others patient’s weight or total daily insulin need based on current treatment, which may be corrected for high HbA1c levels.^56^,^76^ Individualization also implies that automatic adaptation of the individual parameter(s) is required during the course of closed-loop treatment with a time scale of days. Non-automatic adaptation introduces a risk of over- or under-dosing, since patients or health care providers may not (in time) notice the need for adaptation. Automatic adaptation can differ from relatively simple to advanced methods, but should consider the occurrence of hypo- and hyper-glycemic events since these are the precursors of severe adverse events.^8^ Glucose swings typically result from over-dosing and are one sign of insufficient adaptation of the control algorithms to the patient.^79^

In addition, glucose control algorithms should contain multiple specific measures to further mitigate the risk of hypoglycemia.^11^,^23^ Options are to calculate the insulin-on-board to explicitly take the delayed action of insulin into account, to use algorithms that predict hypoglycemia and consequently reduce or stop insulin infusion, or to use pre-programmed basal insulin rates as the starting point for insulin delivery and only cautiously increase these if glucose values increase.^23^,^56^,^62^,^77^ These measures are, however, not expected to be able to prevent hypoglycemia in all daily life situations, because of the prolonged action of insulin and only one-way control is possible with insulin.^34^,^63^,^76^ To further mimic physiologic glucose control and mitigate the risk of hypoglycemia, the use of glucagon may become an important safety measure,^4^,^63^,^76^ especially for fully automated systems for day and night closed-loop glucose control. For successful glucagon action, the insulin-on-board should be taken into account, as high insulin levels at the time of glucagon delivery limits the effect of glucagon.^3^,^13^ Moreover, it should not be possible that control algorithms deliver both insulin and glucagon at the same time.^78^ Before glucagon can be widely used in the artificial pancreas, a glucagon formulation that is stable for at least a week should become available on the market and the effectiveness of repeated glucagon administration has to be assessed.^76^ In a recently published study, Castle *et al.* demonstrated in eight adults with type 1 diabetes that glycogen stores and the hyperglycemic response were maintained after repeated glucagon administration.^12^ At last, alarms should be given to recommend the patient to take carbohydrates in case hypoglycemia does occur.^23^

Furthermore, glucose control algorithms can depend on manual announcements to indicate certain events. Meal announcements are often part of control algorithms, because this enables the delivery of an insulin bolus to minimize postprandial hyperglycemia. Although on average such systems resulted in higher amount of time in range compared to systems without meal announcement, these are not fully automated closed-loop systems and human errors can affect system safety.^23^ Potential errors include forgetting announcements and incorrect carbohydrate estimation which is quite common due to its difficulty,^7^ as well as different food intake than was announced. The associated risks have to be assessed for each system, as these will depend on the specific meal announcement strategy.^17^,^25^,^26^,^30^ These strategies vary from carbohydrate counting 61 to a qualitative announcement of the size and type of meal, e.g. “typical” and “dinner”.^64^

Some final general requirements can be given for control algorithms. It should be possible to safely stop the glucose control for at least 15 min, for example for personal care and maintenance operations, such as replacing a glucose sensor or insulin cartridge. In case of maintenance operations, the glucose control should automatically stop and either automatically restart or prompt the patient to manually restart. In addition, it must be very clear for the patient whether the automated glucose control is functioning or not.^8^ If a patient has to take over the glucose control in case of failure of one or more components, he must be able to see the insulin (and glucagon) delivery history. Furthermore, control algorithms should be able to handle a few missing sensor glucose values, as this is likely to occur, but should not determine control actions if no glucose values are available for more than a certain amount of time which will be dependent upon the control algorithm (typically 10–20 min). If no control actions can be determined by the control algorithms this should result in safe transition to a fallback therapy and alarms should warn the patient.^8^

### Infusion Pump

The infusion pump delivers the amount of insulin (or glucagon) prescribed by the control algorithms. To enable adequate glucose control with an artificial pancreas, this hormone delivery has to be accurate and reliable. At this moment, infusion pumps for subcutaneous insulin administration are used in artificial pancreas systems. Two subtypes are available: the traditional insulin pump that uses an infusion set with relatively long tubing and the patch pump that is directly adhered to the skin and includes a very short (not visible) infusion set. The traditional pumps can suffer from tubing issues, whereas patch pumps can have problems with adherence and the separate controller.^2^ Compared to the previous described artificial pancreas components, little can be found about safety issues for infusion pumps in artificial pancreas systems. We did not find issues with the accuracy of insulin delivery for the artificial pancreas, but issues with infusion set failures and the delivery site are common in insulin pump therapy.^31^ Infusion set kinking, occlusion, leakage or dislocation may result in under-delivery of insulin or glucagon. Furthermore, local tissue alterations, such as lipohypertrophy, edema, and fibrosis may further delay insulin action.^45^ As problems with the hormone delivery can have serious consequences for a patient using an artificial pancreas, subcutaneous infusion needs to become more reliable through better understanding of the physiological processes and developing improved infusion sets.^31^,^53^

At least the following two measures should be included in artificial pancreas systems to ensure safety with current infusion sets. First, to prevent clotting in the tube or catheter, a small ‘maintenance’ bolus should be given if no dose was given for a long period through that infusion set.^32^ Second, timely detection of delivery failures is very important as these may stay unnoticed by the patient for a substantial period of time.^18^,^65^ The software should be able to detect both obstructed delivery (by using feedback from the pump) and delivery without the expected effect on glucose concentration due to e.g. leakage, dislocation or local tissue alterations (based on control actions and feedback from glucose sensors). In case of such event the patient should be warned and instructed to take appropriate actions. Good fixation of the infusion set(s) is important, which can be facilitated by recommending appropriate infusion sets and instruction of the patient.

Additional safety measures should be included that prevent over-dosing due to various software or hardware failures. Feedback from the pump should be obtained when the delivery is finished indicating the status of the delivery and how many units have actually been delivered. If this response is not received in time, based on the calculated time that it should take, the system must determine the status of the insulin delivery by querying the pump, otherwise the insulin-on-board cannot be correctly calculated. If the software is unable to reliably communicate with the pump then closed-loop control must cease and the system must revert to a safe mode of operation. A top safety layer should examine all insulin requests and block or reduce requests that the algorithm deems unsafe. Furthermore, maximum insulin (and glucagon) amounts that may be given per specific time periods should be defined based on the individual settings of the control algorithms.^30^,^72^ In case such a maximum amount has been delivered by the pump, the current dosing should be stopped.

For bi-hormonal artificial pancreas systems, safety measures have to be taken that prevent switching of insulin and glucagon and thus delivery of the wrong hormone. Separate pumps for insulin and glucagon delivery have to be included in the system. These pumps should have separate drivers to reduce the chance of activating the wrong pump. Importantly, it should be impossible to place an insulin cartridge in the glucagon pump or vice versa by the design of the pump chambers. In addition, the connection between the cartridge and infusion set should be different for insulin and glucagon, for example by using Luer-lock and a proprietary connection. An extra measure is that the infusion sets for insulin and glucagon are distinguishable by color, marks or text. In that case the patient has additional visual information about which infusion set is for which hormone, which is especially useful for lengthy tubes, to assure for example that during flushing the right tube will be disconnected from the cannula.

Finally, the use of pre-filled insulin (and glucagon) cartridges is preferred. Compared to cartridges that have to be filled by the patient, this requires less human interaction which reduces the risk of errors.

**Table 1:** Safety of the artificial pancreas components.

Component	Safety aspects	Mitigation measures
Continuous glucose monitor	Inaccuracies, especially during hypoglycemia	MARD <15%
	Moderate (ARD ≥20%) and large (ARD ≥40%) measurement errors	Identify situations with reduced accuracy and take appropriate measures, e.g. sensor redundancy, software measures
	Inaccurate reference glucose for calibration	Calibration only in case of difference between SMBG and sensor; Use of multiple SMBG values
	Sensor delay and background current	Calibration only during stable euglycemia
	Sensor drift	Detection of glucose sensor inaccuracies and failure; Recalibration
	Sensor unavailability due to loss of communication or sensor replacement	Sensor redundancy
Other sensors	Incorrect adaptation of the control algorithms	Systematically determine efficacy and safety of including physiological parameters into control algorithms
	Inaccuracy	Accuracy requirements depend on influence on glucose control algorithms
	Sensor unavailability due to loss of communication or noncompliance of the patient	Demonstrate that unavailability does not comprise safety
Glucose control algorithms	Uncontrolled real-life situations	Design requirements tailored to target population, environment and treatment target; Responsive to changes in glucose trends
	Delayed action of s.c. infused insulin	Specific measures aimed at hypoglycemia risk, e.g. insulin-on-board calculation, use of glucagon
	Variability PK/insulin sensitivity between/within patients	Compensate for short term changes in insulin sensitivity; Individualization and automatic adaptation
	Missing or incorrect manual announcements	Assessment of risks and measures depend on specific announcement strategies
	Missing glucose values	System should be able to handle a few missing glucose values; Safe transition to fallback therapy if system fails
Infusion pump	Infusion set kinking or occlusion	Give maintenance boluses; Detection of obstructed delivery
	Infusion set leakage or dislocation	Good fixation of infusion set; Detection of delivery without the expected effect on glucose
	Software or hardware failures	Guard and prevent overdosing, e.g. use feedback of pump, top safety layer, set maximum doses
	Bihormonal system: switching of insulin and glucagon	Use of separate pumps, different cartridges and infusion set connections
	Human errors with filling cartridges	Use of pre-filled cartridges

(*M*)*ARD* (mean) absolute relative difference, *CGM* continuous glucose monitor, *PK* pharmacokinetics

## Safety of the Artificial Pancreas System

### Combining the Components

The different components of an artificial pancreas have to be combined to form one system. Close cooperation and data exchange between these components are essential.^72^ If one of the main components or its communication is not working properly, the whole system is affected and the automated glucose control will be comprised or interrupted, which increases the risk of over- or under-dosing. To date, wearable artificial pancreas systems used in clinical studies are typically composed of commercially available continuous glucose monitors, insulin pumps and consumer electronics devices, such as a smartphone or tablet, that serve as the platform on which the control algorithms run. This platform also enables communication between the devices and acts as the interface between the system and the user. These separate devices used to construct an artificial pancreas system are as of yet not approved for this particular application. Combining components from different diabetes technology companies is a challenging task as proprietary data and communication protocols are common.^59^ The reliability and security of wireless communication between the components and the accompanying power consumption are considered to be weak points of these artificial pancreas systems and should be solved to increase the safety for use in daily life.^23^,^41^,^69^,^71^ Furthermore, using consumer electronics and its operating system as a medical device like the artificial pancreas raises regulatory questions about safety and reliability, for example about interference of other applications or operating system updates.^58^

The type of configuration chosen has a significant effect upon performance and capabilities of the system. Various groups have pursued different system design philosophies. One approach is to attempt to minimize the risk of system failures by integrating the different components of the artificial pancreas system.^10^,^49^ Fewer separate devices may reduce failures of communication, high power consumption due to wireless communication, unauthorized remote control or access to software, and use or storage of invalid data.^57^ Other groups have chosen a modular approach to system design, which allows the various wirelessly connected components a degree of autonomy operation which is designed to support “graceful degradation” of the system in the event that one or more components or system links fail.^38^ For each separate device using wireless communication the advantages and disadvantages regarding functionality and safety should be evaluated to determine if the risks are acceptable. Furthermore, communication directions and frequency, and data and system security measures should be carefully assessed. Recently, the Diabetes Technology Society released the Cybersecurity Standard for Connected Diabetes Devices, which aims to provide a framework for specifying the security requirements for these devices and how to independently assure that these are met.^39^ The main requirements consider cryptography, secure and authorized communication with devices, and integrity protection of software and data.

An important issue in this discussion is the communication with the glucose sensor(s), as this will likely be wireless using a radio transmitter. The transmitted radio waves do not travel well through water and thus communication can get lost if the body is between the transmitter and receiver. For the artificial pancreas that depends on glucose sensor values during all kind of activities and with different device wear positions, we believe this issue is an important safety constraint which has not yet received sufficient attention in research. The communication losses reported for studies under well controlled circumstances give an indication of the extent of this issue.^54^,^55^ The associated risks and possible mitigation measures will have to be carefully investigated in clinical studies performed under controlled and uncontrolled real-life situations. It may turn out that wired communication (e.g. in combination with the infusion set) or improved wireless communication techniques are indicated.

The device(s) must be designed to have as low power consumption as possible. Battery life should ideally last several days to minimize battery change or charge, although for some systems overnight charging may be possible indicating that battery life of 18–24 h may be sufficient. If chargeable batteries are used, charging should be possible with a widely available connector and adaptor as people may forget to bring their charger. The same applies to replaceable batteries, these should be widely available. A second power consideration is that it has to be assured that simultaneous activation of multiple electronic components that use high power does not lead to tasks not being performed or a device shut down. This might for example be the case for wireless communication together with an active pump. Thirdly, data and settings essential for correct functioning of the control algorithms should be stored in a memory that is not affected by empty or changing batteries (non-volatile memory).

Dedicated operating system and software, developed and tested according to the standard IEC 62304, are typically used to ensure stable and safe functioning of each device. However, the FDA has recently indicated a willingness to consider permitting the use of consumer software such as operating systems for mobile devices such as smartphones. The potential safety issues, e.g. difficulties with software upgrades, of using such a solution will need to be carefully evaluated. Modular software design is recommended as it enables flexibility and facilitates testing and obtaining regulatory approval.^56^ Redundancy of essential elements is a known strategy to increase safety in other processes or systems.^8^ As discussed, this can be applied to the glucose monitoring, but another essential element is the processor. If the processor fails or becomes corrupted, this can be detected with a second ‘safety processor’ that checks or guards essential functions performed by the main processor. This safety processor should activate a safety mode in which the pumps are immediately stopped and the patient is warned with alarms. Moreover, the two processors should preferably be from different manufacturers, to reduce the chance of mutual software or hardware errors.

### Alarms

Alarms are a mitigation measure for faults that are detected by the system but that cannot be solved by the system on its own. Alarms provided by the artificial pancreas or the accompanying remote monitoring application may warn the patient, her relatives, important others or health care providers. The risk reduction due to alarms depends on the effectiveness of the fault detection, how the alarm is given and the reaction of the warned person. A safety issue raised by the alarms itself is that if too many alarms occur, including less important and false alarms, the alarms may be ignored or incorrect action may be taken in response to the alarm.^8^

To enhance safety provided by alarms, the system should contain only a restricted number of alarms, which are the important alarms that require action from the patient. As the artificial pancreas provides automated glucose control, low and high glucose values that can be solved by the system itself should not lead to alarms. In addition, multiple ways of giving an alarm should be included in the system, such as sound, speech, vibration and visual information, and it should be evaluated if each alarm is adequately noticed and understood by the user. Alarms given to others by a remote monitoring application should ideally not be part of the risk mitigation measures of the artificial pancreas; safety should not rely on other devices (including delays or failures in data transmission) and people beside the artificial pancreas and its user.^53^,^60^ However, remote monitoring can be a valuable tool for safety in clinical trials and to gain knowledge about the treatment and device functioning, for example for parents and health care professionals, but also for device development or quality control.^58^

### User Aspects

The patient will have to use the artificial pancreas every day and it is therefore emphasized that user acceptance and interaction play an important role in the design of safe devices for diabetes treatment.^72^,^57^,^60^,^66^ Although glucose control will be automated with an artificial pancreas, the patient has to perform daily maintenance to enable the system to function properly. There will be frequent interaction between the patient and the system, because of the need for SMBG input, sufficiently charged batteries and changes of infusion sets, cartridges and glucose sensors, and to react on alarms. Experienced technical difficulties and interruptions in daily living are considered to be a concern for the acceptance of artificial pancreas systems.^5^

Several measures can be taken to improve the ease of use and acceptance of the system. This will reduce user errors and facilitate truly continuous use by the patients, which increases safety as interruption of the glucose control increases the chance of hypo- and hyperglycemia. For all aspects that require interaction with the user, the patient should be involved in the design using appropriate human factors methods such as usability studies.^66^ Especially the user interface is important, which should be intuitive, guided by clear marks and text, and indicate the status of the different components and required actions.^37^ Mechanical aspects such as device shape, size and connections should also be evaluated. Besides, psychosocial impact of the system needs to be assessed and considered in the different design phases.^5^ The artificial pancreas will be worn during all kinds of activities, so it should ideally be weather-, play- and sport-proof.^72^ Furthermore, the user manual and training of the patient can add to safe use of the artificial pancreas. Both have to focus on the maintenance actions and understanding and recognizing system failures including how to handle the possible alarms. The user manual should not be too extensive and the instruction should be tailored to the patient.

**Table 2:** Safety of the artificial pancreas system

Topic	Safety aspects	Mitigation measures
Combining the components	Reliability and security wireless communication	Integration of devices; Evaluate functionality and safety of wireless communication and take appropriate measures
	Cybersecurity	Specify requirements regarding cryptography, secure and authorized communication, and integrity protection of software and data
	Low batteries	Minimize power consumption to enable battery life of at least multiple days; Use of widely available batteries or charger; Appropriate use of electronic components with high power consumption and storage of essential data
	Reliability operating system and software	Dedicated operating system and software; Modular software design; Safety processor
Alarms	Alarm fatigue	Restricted number of alarms: only alarms that require action from the patient
	No response to alarm	Multiple ways of giving alarms; Evaluate adequacy alarms
User	Low acceptance by patients	Involve user and psychosocial impact in design; Weather-, play-, sport-proof system
	Difficulties in use	Intuitive user-interface that indicates component status and required actions; User manual and training with focus on required actions

## Conclusion

Compared to current diabetes treatment, the artificial pancreas holds promise but adds challenging safety issues because it combines several components into one system and takes over glucose control from the patient. To design a safe artificial pancreas, the configuration and implementation of the different components should be directed by risk management. For safety issues that cannot be sufficiently solved by design, timely detection of faults is necessary to alarm the patient. Prerequisites that enable the control algorithms to provide safe closed-loop glucose control are accurate and reliable input of glucose values, assured hormone delivery and an efficient user interface.

Ongoing and future out-of-hospital and unsupervised studies will teach us more about the occurrence of safety issues and the effectiveness of mitigation measures, but the latter may first have to be demonstrated in controlled studies.^72^ We should, however, remember that it is not possible to guarantee 100% safety and insisting on this will limit innovation, while patients and their families and health care providers are eagerly waiting for the artificial pancreas to become available on the market. Perfect should not become the enemy of good. Therefore, the goal should be to develop an artificial pancreas that is as safe as possible based on current knowledge and technical possibilities, for which direction is given in this review. As glucose control is an evolving process, corrective actions remain possible in case of failures as long as these situations are being noticed by the patient.

The establishment of registries to collect data on patients’ clinical variables, device use and failures may contribute to post-market improvements in safety of artificial pancreas systems. Technological advancements that will likely contribute most to safety are faster acting insulins, more accurate glucose sensors and more reliable wireless communication.

## Abbreviations

CTR: Control-to-range
CTT: Control-to-target
FDA: Food and drug administration
IDE: Investigational device exemption
LGS: Low-glucose suspend
MARD: Mean absolute relative difference
PMA: Premarket approval
SMBG: Self-monitored blood glucose

## References

[1] American Diabetes Association (2015). 6. Glycemic targets. Diabetes Care.

[2] Anhalt H, Bohannon NJ (2010). Insulin patch pumps: their development and future in closed-loop systems. Diabetes Technol. Ther..

[3] Bakhtiani PA, El Youssef J, Duell AK, Branigan DL, Jacobs PG, Lasarev MR, Castle JR, Ward WK (2014). Factors affecting the success of glucagon delivered during an automated closed-loop system in type 1 diabetes. J. Diabetes Complicat..

[4] Bakhtiani PA, Zhao LM, El Youssef J, Castle JR, Ward WK (2013). A review of artificial pancreas technologies with an emphasis on bi-hormonal therapy. Diabetes Obes. Metab..

[5] Barnard KD, Wysocki T, Thabit H, Evans ML, Amiel S, Heller S, Young A, Hovorka R, Angela C (2015). Psychosocial aspects of closed- and open-loop insulin delivery: closing the loop in adults with Type 1 diabetes in the home setting. Diabet. Med..

[6] Beck RW, Tamborlane WV, Bergenstal RM, Miller KM, DuBose SN, Hall CA (2012). The T1D Exchange clinic registry. J. Clin. Endocrinol. Metab..

[7] Bequette BW (2012). Challenges and recent progress in the development of a closed-loop artificial pancreas. Annu. Rev. Control.

[8] Bequette BW (2014). Fault detection and safety in closed-loop artificial pancreas systems. J. Diabetes Sci. Technol..

[9] Bergenstal RM, Klonoff DC, Garg SK, Bode BW, Meredith M, Slover RH, Ahmann AJ, Welsh JB, Lee SW, Kaufman FR (2013). Threshold-based insulin-pump interruption for reduction of hypoglycemia. N. Engl. J. Med..

[10] Blauw H, van Bon AC, Koops R, DeVries JH, PCDIAB Consortium (2016). Performance and safety of an integrated bihormonal artificial pancreas for fully automated glucose control at home. Diabetes Obes. Metab..

[11] Cameron F, Bequette BW, Wilson DM, Buckingham BA, Lee H, Niemeyer G (2011). A closed-loop artificial pancreas based on risk management. J. Diabetes Sci. Technol..

[12] Castle JR, El Youssef J, Bakhtiani PA, Cai Y, Stobbe JM, Branigan D, Ramsey K, Jacobs P, Reddy R, Woods M, Ward WK (2015). Effect of repeated glucagon doses on hepatic glycogen in Type 1 diabetes: implications for a bihormonal closed-loop system. Diabetes Care.

[13] Castle JR, Engle JM, El Youssef J, Massoud RG, Ward WK (2010). Factors influencing the effectiveness of glucagon for preventing hypoglycemia. J. Diabetes Sci. Technol..

[14] Castle JR, Pitts A, Hanavan K, Muhly R, El Youssef J, Hughes-Karvetski C, Kovatchev B, Ward WK (2012). The accuracy benefit of multiple amperometric glucose sensors in people with type 1 diabetes. Diabetes Care.

[15] Castle JR, Ward WK (2010). Amperometric glucose sensors: sources of error and potential benefit of redundancy. J. Diabetes Sci. Technol..

[16] Chassin LJ, Wilinska ME, Hovorka R (2004). Evaluation of glucose controllers in virtual environment: methodology and sample application. Artif. Intell. Med..

[17] Chernavvsky DR, DeBoer MD, Keith-Hynes P, Mize B, McElwee M, Demartini S, Dunsmore SF, Wakeman C, Kovatchev BP, Breton MD (2016). Use of an artificial pancreas among adolescents for a missed snack bolus and an underestimated meal bolus. Pediatr. Diabetes..

[18] Clarke WL, Renard E (2012). Clinical requirements for closed-loop control systems. J. Diabetes Sci Technol..

[19] Colberg SR, Laan R, Dassau E, Kerr D (2015). Physical activity and type 1 diabetes: time for a rewire?. J. Diabetes Sci. Technol..

[20] Damiano ER, McKeon K, El-Khatib FH, Zheng H, Nathan DM, Russell SJ (2014). A comparative effectiveness analysis of three continuous glucose monitors: the navigator, G4 platinum, and enlite. J. Diabetes Sci. Technol..

[21] Dassau E, Palerm CC, Zisser H, Buckingham BA, Jovanovic L, Doyle FJ (2009). In silico evaluation platform for artificial pancreatic beta-cell development—a dynamic simulator for closed-loop control with hardware-in-the-loop. Diabetes Technol. Ther..

[22] Davey RJ, Howe W, Paramalingam N, Ferreira LD, Davis EA, Fournier PA, Jones TW (2013). The effect of midday moderate-intensity exercise on postexercise hypoglycemia risk in individuals with type 1 diabetes. J. Clin. Endocrinol. Metab..

[23] Doyle FJ, Huyett LM, Lee JB, Zisser HC, Dassau E (2014). Closed-loop artificial pancreas systems: engineering the algorithms. Diabetes Care..

[24] Elleri D, Allen JM, Kumareswaran K, Leelarathna L, Nodale M, Caldwell K, Cheng P, Kollman C, Haidar A, Murphy HR, Wilinska ME, Acerini CL, Dunger DB, Hovorka R (2013). Closed-loop basal insulin delivery over 36 hours in adolescents with type 1 diabetes: randomized clinical trial. Diabetes Care.

[25] Elleri D, Biagioni M, Allen JM, Kumareswaran K, Leelarathna L, Caldwell K, Nodale M, Wilinska ME, Haidar A, Calhoun P, Kollman C, Jackson NC, Umpleby AM, Acerini CL, Dunger DB, Hovorka R (2015). Safety, efficacy and glucose turnover of reduced prandial boluses during closed-loop therapy in adolescents with type 1 diabetes: a randomized clinical trial. Diabetes Obes. Metab..

[26] Elleri D, Maltoni G, Allen JM, Nodale M, Kumareswaran K, Leelarathna L, Thabit H, Caldwell K, Wilinska ME, Calhoun P, Kollman C, Dunger DB, Hovorka R (2014). Safety of closed-loop therapy during reduction or omission of meal boluses in adolescents with type 1 diabetes: a randomized clinical trial. Diabetes Obes. Metab..

[27] Frier BM (2014). Hypoglycaemia in diabetes mellitus: epidemiology and clinical implications. Nat. Rev. Endocrinol..

[28] Garcia A, Rack-Gomer AL, Bhavaraju NC, Hampapuram H, Kamath A, Peyser T, Facchinetti A, Zecchin C, Sparacino G, Cobelli C (2013). Dexcom G4AP: an advanced continuous glucose monitor for the artificial pancreas. J. Diabetes Sci. Technol..

[29] Gondhalekar R, Dassau E, Zisser HC, Doyle FJ (2013). Periodic-zone model predictive control for diurnal closed-loop operation of an artificial pancreas. J. Diabetes Sci. Technol..

[30] Haidar A, Farid D, St-Yves A, Messier V, Chen V, Xing D, Brazeau AS, Duval C, Boulet B, Legault L, Rabasa-Lhoret R (2014). Post-breakfast closed-loop glucose control is improved when accompanied with carbohydrate-matching bolus compared to weight-dependent bolus. Diabetes Metab..

[31] Heinemann L, Krinelke L (2012). Insulin infusion set: the Achilles heel of continuous subcutaneous insulin infusion. J. Diabetes Sci. Technol..

[32] Hernando ME, Garcia-Saez G, Martinez-Sarriegui I, Rodriguez-Herrero A, Perez-Gandia C, Rigla M, de Leiva A, Capel I, Pons B, Gomez EJ (2009). Automatic data processing to achieve a safe telemedical artificial pancreas. J. Diabetes Sci. Technol..

[33] Hinshaw L, Dalla Man C, Nandy DK, Saad A, Bharucha AE, Levine JA, Rizza RA, Basu R, Carter RE, Cobelli C, Kudva YC, Basu A (2013). Diurnal pattern of insulin action in type 1 diabetes: implications for a closed-loop system. Diabetes.

[34] Hovorka R (2011). Closed-loop insulin delivery: from bench to clinical practice. Nat. Rev. Endocrinol..

[35] Hovorka R, Elleri D, Thabit H, Allen JM, Leelarathna L, El-Khairi R, Kumareswaran K, Caldwell K, Calhoun P, Kollman C, Murphy HR, Acerini CL, Wilinska ME, Nodale M, Dunger DB (2014). Overnight closed-loop insulin delivery in young people with type 1 diabetes: a free-living, randomized clinical trial. Diabetes Care.

[36] Jacobs PG, El Youssef J, Castle J, Bakhtiani P, Branigan D, Breen M, Bauer D, Preiser N, Leonard G, Stonex T, Ward WK (2014). Automated control of an adaptive bihormonal, dual-sensor artificial pancreas and evaluation during inpatient studies. IEEE Trans. Biomed. Eng..

[37] Keith-Hynes P, Guerlain S, Mize B, Hughes-Karvetski C, Khan M, McElwee-Malloy M, Kovatchev BP (2013). DiAs user interface: a patient-centric interface for mobile artificial pancreas systems. J. Diabetes Sci. Technol..

[38] Keith-Hynes P, Mize B, Robert A, Place J (2014). The diabetes assistant: a smartphone-based system for real-time control of blood glucose. Electronics.

[39] Klonoff DC, Kleidermacher DN (2016). Now is the time for a cybersecurity standard for connected diabetes devices. J. Diabetes Sci. Technol..

[40] Kovatchev BP, Patek SD, Ortiz EA, Breton MD (2015). Assessing sensor accuracy for non-adjunct use of continuous glucose monitoring. Diabetes Technol. Ther..

[41] Kovatchev BP, Renard E, Cobelli C, Zisser HC, Keith-Hynes P, Anderson SM, Brown SA, Chernavvsky DR, Breton MD, Mize LB, Farret A, Place J, Bruttomesso D, Del Favero S, Boscari F, Galasso S, Avogaro A, Magni L, Di Palma F, Toffanin C, Messori M, Dassau E, Doyle FJ (2014). Safety of outpatient closed-loop control: first randomized crossover trials of a wearable artificial pancreas. Diabetes Care.

[42] Kowalski AJ (2009). Can we really close the loop and how soon? Accelerating the availability of an artificial pancreas: a roadmap to better diabetes outcomes. Diabetes Technol. Ther..

[43] Kropff J, Bruttomesso D, Doll W, Farret A, Galasso S, Luijf YM, Mader JK, Place J, Boscari F, Pieber TR, Renard E, DeVries JH (2014). Accuracy of two continuous glucose monitoring systems: a head-to-head comparison under clinical research centre and daily life conditions. Diabetes Obes Metab..

[44] Kropff J, Del Favero S, Place J, Toffanin C, Visentin R, Monaro M, Messori M, Di Palma F, Lanzola G, Farret A, Boscari F, Galasso S, Magni P, Avogaro A, Keith-Hynes P, Kovatchev BP, Bruttomesso D, Cobelli C, DeVries JH, Renard E, Magni L, A. P. h. consortium (2015). 2 month evening and night closed-loop glucose control in patients with type 1 diabetes under free-living conditions: a randomised crossover trial. Lancet Diabetes Endocrinol..

[45] Kudva YC, Carter RE, Cobelli C, Basu R, Basu A (2014). Closed-loop artificial pancreas systems: physiological input to enhance next-generation devices. Diabetes Care.

[46] Leelarathna L, Nodale M, Allen JM, Elleri D, Kumareswaran K, Haidar A, Caldwell K, Wilinska ME, Acerini CL, Evans ML, Murphy HR, Dunger DB, Hovorka R (2013). Evaluating the accuracy and large inaccuracy of two continuous glucose monitoring systems. Diabetes Technol. Ther..

[47] Ly TT, Breton MD, Keith-Hynes P, De Salvo D, Clinton P, Benassi K, Mize B, Chernavvsky D, Place J, Wilson DM, Kovatchev BP, Buckingham BA (2014). Overnight glucose control with an automated, unified safety system in children and adolescents with type 1 diabetes at diabetes camp. Diabetes Care.

[48] Ly TT, Nicholas JA, Retterath A, Lim EM, Davis EA, Jones TW (2013). Effect of sensor-augmented insulin pump therapy and automated insulin suspension vs standard insulin pump therapy on hypoglycemia in patients with type 1 diabetes: a randomized clinical trial. JAMA.

[49] Ly TT, Roy A, Grosman B, Shin J, Campbell A, Monirabbasi S, Liang B, von Eyben R, Shanmugham S, Clinton P, Buckingham BA (2015). Day and night closed-loop control using the integrated medtronic hybrid closed-loop system in type 1 diabetes at diabetes camp. Diabetes Care.

[50] Mauseth R, Hirsch IB, Bollyky J, Kircher R, Matheson D, Sanda S, Greenbaum C (2013). Use of a “fuzzy logic” controller in a closed-loop artificial pancreas. Diabetes Technol. Ther..

[51] McMahon SK, Ferreira LD, Ratnam N, Davey RJ, Youngs LM, Davis EA, Fournier PA, Jones TW (2007). Glucose requirements to maintain euglycemia after moderate-intensity afternoon exercise in adolescents with type 1 diabetes are increased in a biphasic manner. J. Clin. Endocrinol. Metab..

[52] Nimri R, Muller I, Atlas E, Miller S, Fogel A, Bratina N, Kordonouri O, Battelino T, Danne T, Phillip M (2014). MD-logic overnight control for 6 weeks of home use in patients with type 1 diabetes: randomized crossover trial. Diabetes Care.

[53] Nimri R, Phillip M (2014). Artificial pancreas: fuzzy logic and control of glycemia. Curr. Opin. Endocrinol. Diabetes Obes..

[54] O’Grady MJ, Retterath AJ, Keenan DB, Kurtz N, Cantwell M, Spital G, Kremliovsky MN, Roy A, Davis EA, Jones TW, Ly TT (2012). The use of an automated, portable glucose control system for overnight glucose control in adolescents and young adults with type 1 diabetes. Diabetes Care.

[55] Oron T, Farfel A, Muller I, Miller S, Atlas E, Nimri R, Phillip M (2014). A remote monitoring system for artificial pancreas support is safe, reliable, and user friendly. Diabetes Technol. Ther..

[56] Patek SD, Magni L, Dassau E, Karvetski C, Toffanin C, De Nicolao G, Del Favero S, Breton M, Man CD, Renard E, Zisser H, Doyle FJ, Cobelli C, Kovatchev BP, G. International Artificial Pancreas Study (2012). Modular closed-loop control of diabetes. IEEE Trans. Biomed. Eng..

[57] Paul N, Kohno T, Klonoff DC (2011). A review of the security of insulin pump infusion systems. J. Diabetes Sci. Technol..

[58] Peyser T, Dassau E, Breton M, Skyler JS (2014). The artificial pancreas: current status and future prospects in the management of diabetes. Ann. N. Y. Acad. Sci..

[59] Picton PE, Yeung M, Hamming N, Desborough L, Dassau E, Cafazzo JA (2013). Advancement of the artificial pancreas through the development of interoperability standards. J. Diabetes Sci. Technol..

[60] Place J, Robert A, Ben Brahim N, Keith-Hynes P, Farret A, Pelletier MJ, Buckingham B, Breton M, Kovatchev B, Renard E (2013). DiAs web monitoring: a real-time remote monitoring system designed for artificial pancreas outpatient trials. J. Diabetes Sci Technol..

[61] Reddy M, Herrero P, El Sharkawy M, Pesl P, Jugnee N, Thomson H, Pavitt D, Toumazou C, Johnston D, Georgiou P, Oliver N (2014). Feasibility study of a bio-inspired artificial pancreas in adults with type 1 diabetes. Diabetes Technol. Ther..

[62] Revert A, Garelli F, Pico J, De Battista H, Rossetti P, Vehi J, Bondia J (2013). Safety auxiliary feedback element for the artificial pancreas in type 1 diabetes. IEEE Trans. Biomed. Eng..

[63] Russell SJ (2015). Progress of artificial pancreas devices towards clinical use: the first outpatient studies. Curr. Opin. Endocrinol. Diabetes Obes..

[64] Russell SJ, El-Khatib FH, Sinha M, Magyar KL, McKeon K, Goergen LG, Balliro C, Hillard MA, Nathan DM, Damiano ER (2014). Outpatient glycemic control with a bionic pancreas in type 1 diabetes. N. Engl. J. Med..

[65] Sanchez RM (2013). The critical path from pump to pancreas: the impact of FDA regulation on the development of a closed-loop diabetes management system. Food Drug Law J..

[66] Schaeffer NE (2012). The role of human factors in the design and development of an insulin pump. J. Diabetes Sci. Technol..

[67] Schiavon M, Dalla Man C, Kudva YC, Basu A, Cobelli C (2013). In silico optimization of basal insulin infusion rate during exercise: implication for artificial pancreas. J. Diabetes Sci. Technol..

[68] Stenvers DJ, DeVries JH, la Fleur SE (2013). What’s the Time? Does the Artificial Pancreas Need to Know?. Diabetes.

[69] Tauschmann M, Hovorka R (2014). Insulin pump therapy in youth with type 1 diabetes: toward closed-loop systems. Expert Opin. Drug Deliv..

[70] Thabit H, Hovorka R (2014). Bringing closed-loop home: recent advances in closed-loop insulin delivery. Curr. Opin. Endocrinol. Diabetes Obes..

[71] Thabit H, Tauschmann M, Allen JM, Leelarathna L, Hartnell S, Wilinska ME, Acerini CL, Dellweg S, Benesch C, Heinemann L, Mader JK, Holzer M, Kojzar H, Exall J, Yong J, Pichierri J, Barnard KD, Kollman C, Cheng P, Hindmarsh PC, Campbell FM, Arnolds S, Pieber TR, Evans ML, Dunger DB, Hovorka R, A. P. Consortium and A. P. h. Consortium (2015). Home use of an artificial beta cell in type 1 diabetes. N Engl J Med..

[72] The Content of Investigational Device Exemption (IDE) and Premarket Approval (PMA) Applications for Artificial Pancreas Device Systems. Center for Devices and Radiological Health: U.S. Department of Health and Human Services, Food and Drug Administration, 2012.

[73] Turksoy K, Quinn LT, Littlejohn E, Cinar A (2014). An integrated multivariable artificial pancreas control system. J. Diabetes Sci. Technol..

[74] van Bon AC, Luijf YM, Koebrugge R, Koops R, Hoekstra JB, Devries JH (2014). Feasibility of a portable bihormonal closed-loop system to control glucose excursions at home under free-living conditions for 48 hours. Diabetes Technol. Ther..

[75] Visentin R, Dalla Man C, Kudva YC, Basu A, Cobelli C (2015). Circadian variability of insulin sensitivity: physiological input for in silico artificial pancreas. Diabetes Technol. Ther..

[76] Ward WK, Castle JR, El Youssef J (2011). Safe glycemic management during closed-loop treatment of type 1 diabetes: the role of glucagon, use of multiple sensors, and compensation for stress hyperglycemia. J. Diabetes Sci. Technol..

[77] Wilinska ME, Budiman ES, Taub MB, Elleri D, Allen JM, Acerini CL, Dunger DB, Hovorka R (2009). Overnight closed-loop insulin delivery with model predictive control: assessment of hypoglycemia and hyperglycemia risk using simulation studies. J. Diabetes Sci. Technol..

[78] Zisser H (2011). Clinical hurdles and possible solutions in the implementation of closed-loop control in type 1 diabetes mellitus. J. Diabetes Sci. Technol..

[79] Zisser H, Renard E, Kovatchev B, Cobelli C, Avogaro A, Nimri R, Magni L, Buckingham BA, Chase HP, Doyle FJ, Lum J, Calhoun P, Kollman C, Dassau E, Farret A, Place J, Breton M, Anderson SM, Dalla Man C, Del Favero S, Bruttomesso D, Filippi A, Scotton R, Phillip M, Atlas E, Muller I, Miller S, Toffanin C, Raimondo DM, De Nicolao G, Beck RW, G. Control to Range Study (2014). Multicenter closed-loop insulin delivery study points to challenges for keeping blood glucose in a safe range by a control algorithm in adults and adolescents with type 1 diabetes from various sites. Diabetes Technol. Ther..

